# Barbell Technique for Three-Dimensional Bone Augmentation

**DOI:** 10.1155/2023/4180372

**Published:** 2023-11-09

**Authors:** Marcelo Pereira Nunes, Luís Guilherme Scavone de Macedo, Mauro Pedrine Santamaria, João Carlos Ribeiro, Peter Karyen Moy, André Antonio Pelegrine

**Affiliations:** ^1^Proimperio Institute, São Paulo, Brazil; ^2^Division of Implant Dentistry, São Leopoldo Mandic Dental School, Campinas, Brazil; ^3^College of Dentistry, University of Kentucky, Lexington, USA; ^4^Department of Oral & Maxillofacial Surgery, University of California, Los Angeles, USA

## Abstract

**Introduction:**

Appositional bone augmentation is considered a challenging surgical problem to correct for the deficient alveolar ridge. To overcome this challenge, a novel concept was recently published called “Barbell Technique.” This technique has been used more commonly for horizontal bone augmentation. To our knowledge, this is the first report on using the Barbell Technique for vertical bone augmentation. *Case Report*. This report describes and demonstrates the clinical feasibility of the use of this concept in the reconstruction of a tridimensional alveolar ridge defect in the anterior maxilla. Due to the severity of the defect, both hard and soft tissue deficiencies required augmentation. The first surgery involved a soft tissue grafting procedure while in the second surgical procedure, hard tissue augment was performed using the Barbell device to provide both vertical and horizontal support for the hard tissue graft. The donor material consisted of equal volume of xenograft and autogenous bone used to fill the defect and covered with a collagen barrier membrane. After a healing period of 9 months, the site was reopened. Bone formation clinically verified the correction of alveolar bone contour and volume permitted placement of two titanium implants after the removal of Barbell device.

**Conclusion:**

This case report demonstrates successful vertical and horizontal bone augmentation of a critical size defect in the anterior maxilla, correcting both hard and soft tissue contours, and providing the tissues needed to support dental implants in the anterior maxilla.

## 1. Background

One of the most challenging surgical procedures in the field of implant dentistry is the vertical appositional bone reconstruction [1]. A recent systematic review of the literature showed that guided bone regeneration (GBR) technique is the most predictable, achieving a better vertical bone gain when compared with bone block, as well as fewer complications when compared with bone block and osseous distraction techniques [1]. The idea of the creation of a secluded space to allow the protected migration of osteoblasts was established [2]. The concept of GBR is based on the biologic process of excluding cells that will form soft tissue but permitting initial colonization of vital cells that may initiate hard tissue formation. Using barriers to permit certain desirable cells to gain access to the alveolar defect and excluding cells that form other tissues may enhance the total volume of bone regeneration.

Preclinical studies on a GBR model have shown that block bone substitutes resulted in less bone ingrowth in comparison to a particulate graft. However, after correction of hard and soft tissue ridge contours, bone blocks seem to render more favorable outcomes [3]. A recent randomized clinical trial evaluated the histological outcomes after GBR of peri-implant defects, comparing particulate and block xenografts [4]. This study showed a higher level of bone formation when particulate donor material was used (25.2% for the particulate compared with 11.5% for the block material), after 6 months of healing. According to the authors, a possible explanation for this result could be a higher osteoconductivity due to the differences in the macrostructure of the particulate graft material compared to the block graft material [4]. The particulate biomaterial provided more spacial room between particles, thus permitting more ingrowth of new blood vessels (neovascularity) and new bone. When comparing the same volume with both materials (particulate versus block), the surface area of the particulate graft is higher, which theoretically contribute to improving new bone incorporation. However, the block graft material is a more solidified structure, which may be beneficial in providing stability, especially in large reconstructions requiring appositional bone growth.

To overcome the lack of structural stability with particulate donor materials, the GBR concept for appositional bone augmentation using particulate materials requires the use of form-stable devices (e.g., titanium-reinforced, nonresorbable membranes, or titanium meshes) to withstand the compressive forces from soft tissue healing. Nonrigid, resorbable membranes (e.g., collagen membranes) may collapse in this scenario. However, assuming that resorbable membranes are hydrophilic while nonresorbable membranes/meshes are hydrophobic, the hydrophilicity feature could hypothetically minimize postoperative complications related to nonresorbable membranes such as exposure [5], more frequent occurrence rate of infections, and other adverse events with wound healing [5, 6]. The purpose of this report is to introduce a technique to provide support to the collagen membrane and stability for particulate donor graft material. In this report, a new technique for GBR, using an internal tissue tenting device together with a resorbable membrane and particulate bone graft material, is described. This technique was recently reported and named Barbell Technique [7]. In the first report describing this technique [7], a horizontal bone gain of 6.81 ± 1.33 mm was achieved, resulting in augmentation of both the buccal and palatal sides. Originally, the Barbell Technique was used exclusively for horizontal bone augmentation. However, the objective of this case report was to show and discuss, for the first time, the use of the Barbell Technique for appositional vertical bone augmentation.

## 2. Clinical Presentation

**Figure 1:**
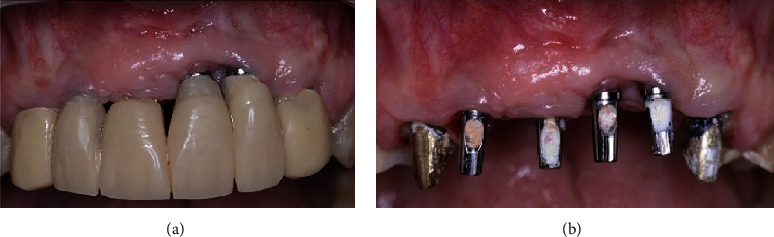
Initial clinical view before (a) and after (b) prosthesis removal. Note the deficient soft tissue contours and unacceptable aesthetics.

A 59-year-old female was referred to the Department of Implant Dentistry at Faculdade de Odontologia, São Leopoldo Mandic, Brazil, for treatment, complaining of poor aesthetics when she smiled. On clinical examination, the presence of four osseointegrated implants was found in the four maxillary incisor sites. The soft tissue contour was irregular, and the implants were poorly positioned (Figures 1(a) and 1(b)). The patient was informed of the implants' poor clinical positions and treatment options and signed an informed consent describing the risks versus benefits before starting the treatment.

**Figure 2:**
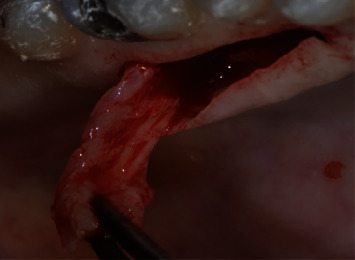
Connective tissue harvesting from the palate.

**Figure 3:**
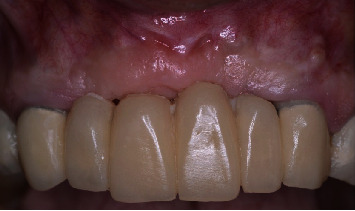
Clinical result after implants were removed, connective tissue graft to augment soft tissue deficiency, and a fixed provisional prosthesis delivered.

The decision was made to remove the implants and reconstruction of the deficient alveolar ridge before new implant placement. After the implants were removed, a healing period of 2 months was permitted, followed by soft tissue grafting. The connective tissue was harvested from the palate, between the first bicuspid and molar area, using the subepithelial connective tissue graft harvesting technique (Figure 2). The aesthetics was improved substantially with a new fixed provisional prosthesis (Figure 3).

## 3. Case Report

**Figure 4:**
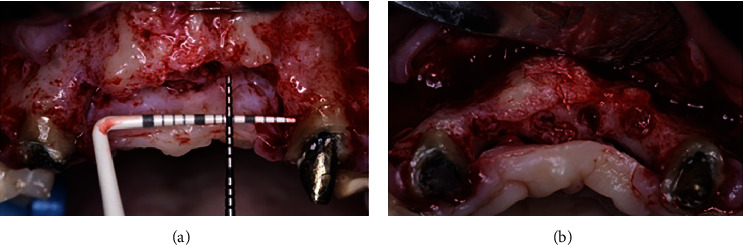
Hard tissue defect from frontal (a) and occlusal (b) views.

**Figure 5:**
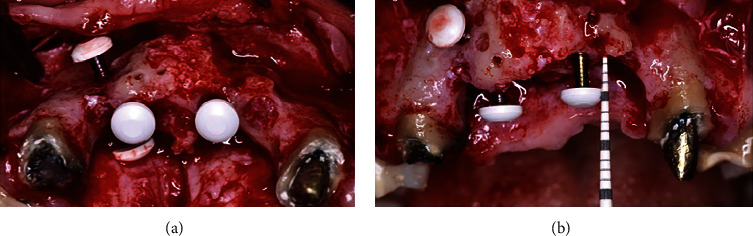
Barbell tenting screw positions from occlusal (a) and frontal (b) views. Note the use of two PEEK caps in the horizontally positioned tenting screw (a) and only one PEEK cap in the vertically positioned tenting screws (b).

**Figure 6:**
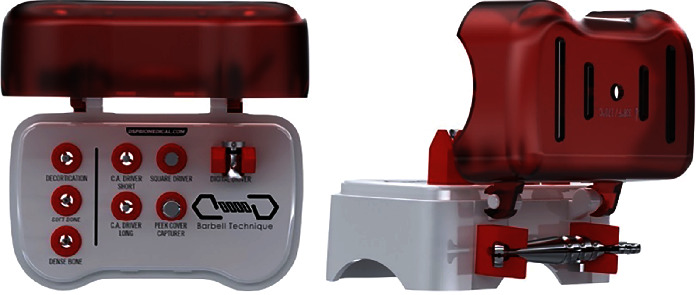
Barbell Technique surgical kit containing various components needed to deliver tenting screws.

After 3 months of healing for the soft tissue graft, the site was reentered to perform the bone graft augmentation procedure. After local anesthesia was applied, a horizontal crestal incision through the keratinized mucosa was performed using a 15C surgical blade and two vertical releasing incisions (mesial and distal), and a mucoperiosteal flap was reflected. After the exposure of the alveolar ridge (Figures 4(a) and 4(b)), three Barbell tenting devices were positioned, one horizontally and two vertically (Figures 5(a) and 5(b)). For this purpose, a surgical kit specifically designed for the Barbell Technique was used (Figures 6(a) and 6(b)). Three Barbell tenting screws were used, two 10 mm, and one 8 mm. The length of the screws was determined by the amount of bone augmentation needed. To prepare the recipient bed for the tenting screws, a soft bone drill was used. The screws were placed into the custom tenting screw carrier and delivered to the recipient bed, and small perforations were created using the decortication drill contained in the Barbell surgical kit to improve blood flow and provide necessary nutrients to the graft site. The horizontally positioned Barbell was placed in the site where the horizontal bone augmentation was required (i.e., the lateral incisor area). The two vertically positioned tenting screws were placed in the middle of the four missing teeth's segment. Once the tenting screws were positioned, the PEEK capsules were connected to the one exposed end of the vertically positioned tenting screws and to both ends of the horizontally positioned tenting screw. The PEEK capsules of the vertically positioned Barbell screws were placed at a similar height to each other and the height of the residual crest along the mesial aspect of each canine. The capsules of the horizontally positioned Barbell screws were placed slightly beyond the buccal and palatal bone walls of the adjacent canines. The three tenting screws were carefully positioned to provide the necessary soft tissue support to prevent soft tissue collapse and to maximize the total volume of newly formed bone.

**Figure 7:**
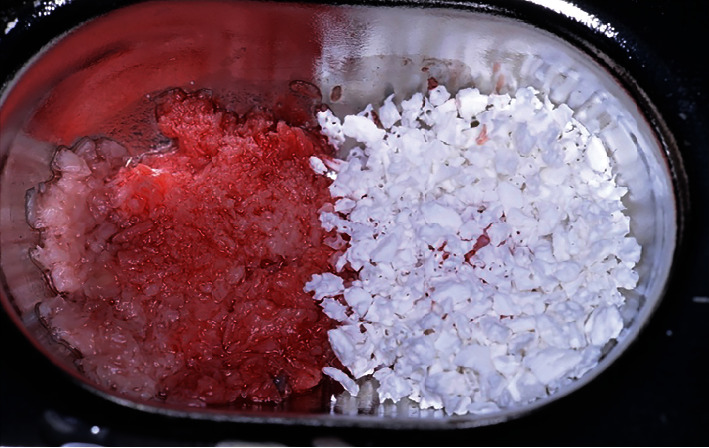
Bone graft materials used in the bone augmentation surgery (autograft on the left side and xenograft on the right side).

**Figure 8:**
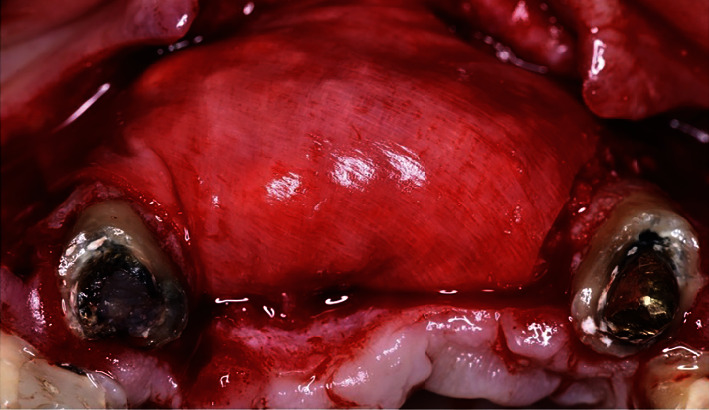
Collagen membrane positioned over the mixed bone graft.

**Figure 9:**
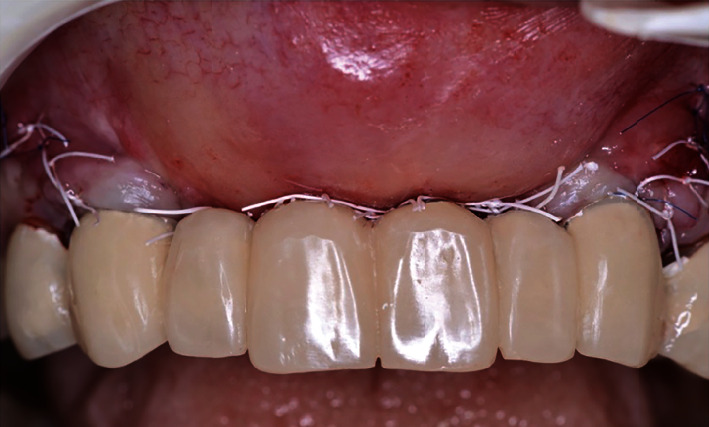
Immediate postoperative view.

A mix of autogenous bone was harvested from the posterior mandible using a scraper (Mx-Grafter, Maxilon Laboratories Inc., Amherst, MA, USA) and a xenograft (Bio-Oss, Geistlich Biomaterials, Wolhusen, Switzerland) in a 1 : 1 ratio (Figure 7). The graft material mixture was used to fill the bone defect and covered with a collagen resorbable membrane (Bio-Gide, Geistlich Biomaterials, Wolhusen, Switzerland) (Figure 8) for guided bone regeneration. The soft tissue flap was closed using horizontal mattress sutures to stabilize the membrane and resist tension and single interrupted 4/0 PTFE sutures (Osteogenics, Lubbock, TX, USA) to achieve primary closure (Figure 9).

## 4. Clinical Outcomes

**Figure 10:**
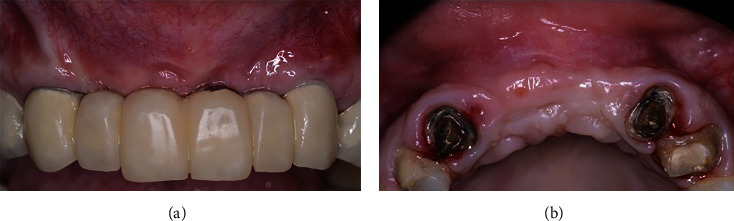
Eight-month postoperative view in frontal (a) and occlusal (b) views. Note the improvement of the tissue contours.

**Figure 11:**
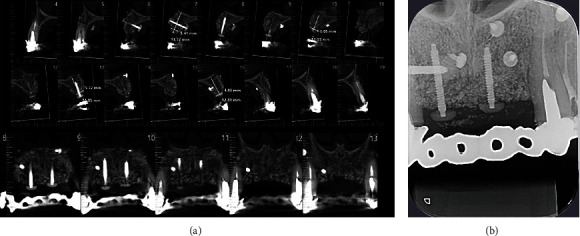
Tomographic (a) and periapical radiographic (b) images, showing improvement of bone contour and volume.

**Figure 12:**
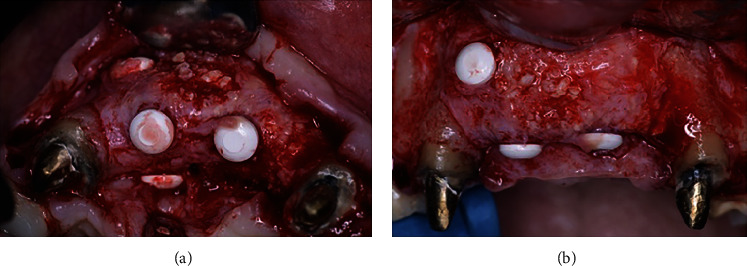
Site reopened after 9 months of healing from occlusal (a) and frontal (b) views. Note the level of bone augmentation achieved.

**Figure 13:**
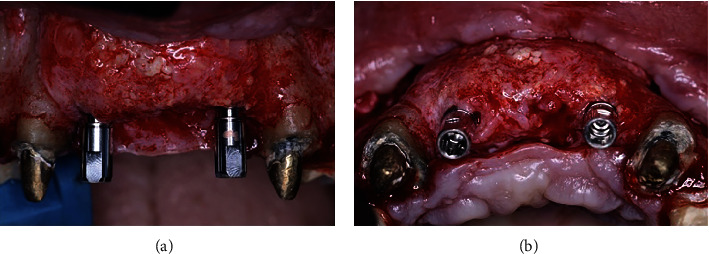
Implants placed with frontal (a) and occlusal (b) views.

After 9 months, the soft tissue contour had improved (Figures 10(a) and 10(b)) and radiologic examination showed improvement in total bone volume (Figures 11(a) and 11(b)). After soft tissue flap reflection, adequate vertical bone regeneration was identified (Figures 12(a) and 12(b)). The Barbell devices were removed by using the same instruments used for their installing, and the two implants were placed at the lateral incisor positions (Figures 13(a) and 13(b)), in accordance with the prosthetic planning. Both implants achieved adequate primary stability, with insertion torque higher than 35 N.cm, and an immediate provisional prosthesis was installed to immediately load the implants.

**Figure 14:**
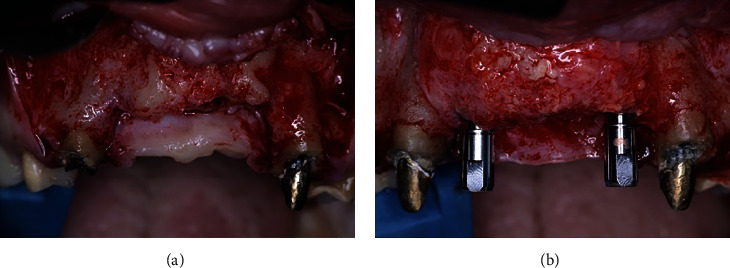
Frontal view at baseline (a) and 9-month postsimultaneous horizontal and vertical augmentation (b).

**Figure 15:**
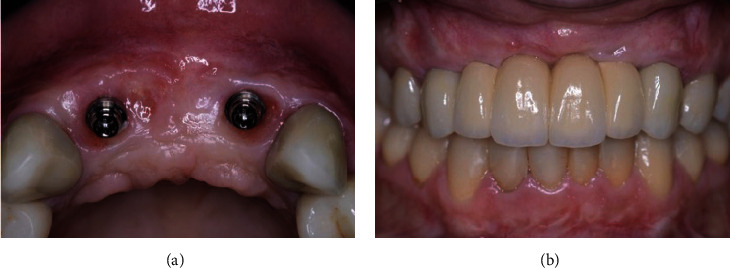
Occlusal view after implant osseointegration and soft tissue healing (a) and a frontal view of the final fixed prosthesis (b).

A comparison between baseline and 9 months after bone augmentation using the Barbell Technique showed the amount of bone regenerated and improved contours of the maxillary anterior ridge (Figures 14(a) and 14(b)). Two implants were installed in the anterior maxilla allowing rehabilitation with a 4-unit fixed prosthesis after 4 months. The 18-month follow-up showed good soft tissue contours and improved aesthetic results (Figures 15(a) and 15(b)).

## 5. Discussion

Several techniques have been used for vertical bone augmentation of the deficient alveolar ridge. Traditionally, GBR using a particulate bone graft (autograft alone or mixed with a bone substitute biomaterial) and a structured barrier membrane, such as a titanium-reinforced membrane, supports soft tissue and prevents compression and collapse of the soft tissue flap during healing. The GBR technique is considered the gold standard for appositional bone augmentation [8]. However, a drawback with the use of a titanium-reinforced membrane is the exposure rate with these membranes when compared with pure collagen membranes. The higher exposure rate results in higher occurrence rate for infections and adverse events with wound healing [5]. The resorbable membrane has the ability to merge with the host tissues due to hydrophilicity properties and has a rapid resorption rate when an exposure of the collagen membrane occurs. This reduces the risk of infection [8]. Resorbable membranes do have a disadvantage when used in appositional reconstruction related to their unfavorable mechanical properties. The lack of stiffness with the pure collagen membranes can lead to collapse of soft tissue into the hard tissue defect, displacing the particulate bone graft material. Therefore, the surgical technique of choice including the use of an internal device that resists tissue compression (e.g., Barbell Technique) together with resorbable membranes should be considered. The disadvantage of a resorbable collagen membrane (i.e., lack of 3D stability) may be overcome with the use of tenting screws provided in the surgical kit.

It is well established that structured grafts (i.e., bone blocks) are beneficial for gaining bone volume as they are more stable. However, from a biological point of view, histomorphometric studies show that particulate bone grafts become more vascularized and incorporated [3], as the spacing between the particles of graft material permits ingrowth of blood vessels bringing in nutrients to enhance new bone formation. The use of a device that prevents soft tissue collapse (e.g., Barbell Technique) should help to overcome the disadvantage of a particulate bone graft (lack of structure), while maintaining its biologic advantages. Moreover, when a particulate graft is used, a 1 : 1 mix between autografts and xenografts provides an advantage as the autograft can provide the osteogenic factors while the xenograft provides more stability over time, helping to overcome the shrinkage tendency of autografts [9]. According to some authors [9, 10], the use of GBR and 1 : 1 ratio of particulate xenograft and autogenous bone for the reconstruction of severe defects showed promising results.

In the present case report, the use of GBR with the Barbell Technique was successful in reconstruction of a tridimensional alveolar ridge defect in the anterior maxilla. Based on literature [1, 11], GBR is commonly preferred over distraction osteogenesis as it allows simultaneous vertical and horizontal augmentation and has fewer complications. GBR success rate is dependent on the ability to provide primary closure of the soft tissue wound, initiation of angiogenesis, donor graft material stability, and proper space maintenance, excluding non-bone-forming cells [12]. The present clinical case used an absorbable collagen membrane for cell guidance used in conjunction with Barbell devices (titanium tenting screws) and a mixture of particulate, autograft, and xenograft. This permitted meeting all of the requirements that is needed for successful regeneration of new bone. The unique characteristic of the Barbell tenting screws allowed for tridirectional horizontal and vertical bone augmentation to effectively achieve tridimensional bone regeneration in this clinical case.

The Barbell Technique is a novel approach for appositional bone reconstruction, based on sound biological and mechanical concepts. There are no comparative studies analyzing the results achieved with the Barbell Technique compared with other surgical approaches for appositional bone reconstruction. The findings of a technical note published in 2020 by Pelegrine et al. [7] showed, in horizontal bone reconstruction, a mean bone volume gain of 6.81 ± 1.33 mm, which is higher than the average volume gained from other surgical techniques published in the scientific literature to manage defects of the alveolar ridge (3.71 ± 0.24 mm), according to a systematic review and meta-analysis performed [13]. However, it is important to state that the magnitude of the horizontal loss may have influence in the decision-making in selection of the surgical technique and clinical outcomes of these studies. In this regard, Pelegrine et al. [14] published a guideline for clinical situations requiring horizontal augmentation and named it the HAC classification (the acronym for horizontal alveolar changes classification). Briefly, the atrophic sites were categorized in four groups, according with the level of bone loss: (a) HAC 1: slight bone resorption with no need for bone reconstruction due to the possibility of immediate implant placement in an ideal position without grafting; (b) HAC 2: slight bone resorption with minor need for bone reconstruction, which can be done with the use of an osteoconductive biomaterial because of the presence of cancellous bone between the cortical buccal and palatal/lingual bone plates. It also allows for a single surgical approach, with immediate implant placement; (c) HAC 3: moderate bone resorption, but still with remaining cancellous bone at the residual alveolar site, requiring just the use of an osteoconductive biomaterial for reconstruction. However, a two-stage surgical approach is usually needed: firstly, just the bone graft procedure and, a few months later, the implant; and (d) HAC 4: severe bone resorption, with no remaining cancellous bone at the residual alveolar site, requiring the use of an osteoconductive, osteoinductive, and osteogenic material (i.e., autogenous bone graft or bone tissue engineering with live cell transplant or bone inductive proteins). As the required bone reconstruction is significant, a two-stage surgical approach is imperative.

Taking this knowledge into account, in the study published by Pelegrine et al. [7], half of the defects demonstrated severe bone resorption resulting in a knife-edge ridge and void of cancellous bone and requiring major bone augmentation (i.e., HAC 4) [14]. This could explain the large difference between the two studies. It should also be noted that the current surgical technique does not allow horizontal augmentation in a bidirectional manner, due to the difficulty grafting the palatal/lingual aspect of the alveolar deficiency (this is overcome using the Barbell Technique). In this regard, Macedo et al. [15] recently showed a horizontal gain of 4.45 ± 0.75 mm, specifically in moderate bone resorption, but still with remaining cancellous bone at the residual alveolar site (i.e., HAC 3), when Barbell Technique is used. The Barbell Technique has produced results for horizontal augmentation that were higher than the average volume of bone gained as stated in the scientific literature.

The Barbell Technique is a derivative of the tent pole technique and is the first surgical technique that allows for bidirectional horizontal bone augmentation in a predictable manner [7], and the device and instrumentation were designed just for the proposed technique. The surgical kit contains rounded PEEK capsules specifically created to prevent compression of the particulate graft material and allow for tissue formation and integration. PEEK is highly biocompatible, increasing adhesion and viability and permitting proliferation of osteoblasts and gingival fibroblasts compared with titanium implant material [16]. Perhaps, the lack of some required characteristics limits the tent pole technique to small bone reconstructions [17], even though some authors in 2021 have shown significant bone augmentation in three clinical cases by using this technique. However, the clinical cases contained one/two wall, tooth defects [18]. In the authors' opinion, Barbell Technique is at least as easy as the tent pole technique and much easier if compared with bone blocks, titanium membranes/meshes, and osseous distraction techniques. At the same time, it is important to note that all appositional bone reconstruction techniques require optimal soft tissue management to achieve primary wound closure, and therefore, the performance of a highly skilled surgeon is imperative.

Although the results in the present case demonstrated that the use of Barbell Technique was successful for both horizontal and vertical bone reconstructions, future studies should be conducted to analyze the long-term stability of this novel approach and compare it with other surgical techniques. There is a limitation with this technique, specifically with fully edentulous patients demonstrating severe atrophy of the alveolar ridge. The PEEK capsules and the particulate bone graft used cannot withstand the compressive forces from the full denture if worn during the healing time. However, this new technique has several advantages that are seen when compared with other surgical techniques commonly used for horizontal and vertical augmentation of the resorbed alveolar ridge with contour deficiencies.

## 6. Summary

- Why is this case new information? This case presents a novel approach and device to gain both vertical and horizontal ridge augmentation simultaneously.
- What are the keys to successful management of this case? The new device prevents soft tissue compression over the grafted area and the displacement of bone particles.
- What are the primary limitations to success in this case? Operator inexperience; soft tissue manipulation is required to gain primary closure.

## 7. Conclusion

In the present case report, the use of Barbell Technique was successful with bone regeneration in a tridimensional alveolar ridge defect of the anterior maxilla, allowing for complete rehabilitation of a severely compromised alveolar ridge with an aesthetic implant-supported prosthesis.

## Data Availability

All data will be made available upon request to the corresponding author.

## Additional Points

*Key Finds*. This is the first case report to describe a vertical bone augmentation using the new device and concept called Barbell Technique.
